# The Cytotoxic Effects of Low Intensity Visible and Infrared Light on Human Breast Cancer (MCF7) cells

**DOI:** 10.5936/csbj.201303015

**Published:** 2013-10-20

**Authors:** P Peidaee, N Almansour, R Shukla, E Pirogova

**Affiliations:** a School of Electrical and Computer Engineering, RMIT University, Melbourne, Australia; b Department of Biotechnology and Environmental Biology, School of Applied Sciences, RMIT University, Australia; c Centre for Advanced Materials and Industrial Chemistry, School of Applied Sciences, RMIT University, Australia; d Health Innovation Research Institute, RMIT University, Australia

**Keywords:** Electromagnetic Radiation (EMR), Lactate dehydrogenase (LDH) Assay, PrestoBlue™ Assay, Light microscopy, Far Infrared wavelength exposure, Visible light exposure

## Abstract

A concept of using low intensity light therapy (LILT) as an alternative approach to cancer treatment is at early stages of development; while the therapeutic effects of LILT as a non-invasive treatment modality for localized joint and soft tissue wound healing are widely corroborated. The LEDs-based exposure system was designed and constructed to irradiate the selected cancer and normal cells and evaluate the biological effects induced by light exposures in visible and infrared light range. In this study, human breast cancer (MCF7) cells and human epidermal melanocytes (HEM) cells (control) were exposed to selected far infrared light (3400nm, 3600nm, 3800nm, 3900nm, 4100nm and 4300nm) and visible and near infrared wavelengths (466nm, 585nm, 626nm, 810nm, 850nm and 950nm). The optical intensities of LEDs used for exposures were in the range of 15µW to 30µW. Cellular morphological changes of exposed and sham-exposed cells were evaluated using light microscopy. The cytotoxic effects of these low intensity light exposures on human cancer and normal cell lines were quantitatively determined by Lactate dehydrogenase (LDH) cytotoxic activity and PrestoBlue™ cell viability assays. Findings reveal that far-infrared exposures were able to reduce cell viability of MCF7 cells as measured by increased LDH release activity and PrestoBlue™ assays. Further investigation of the effects of light irradiation on different types of cancer cells, study of possible signaling pathways affected by electromagnetic radiation (EMR) and *in vivo* experimentation are required in order to draw a firm conclusion about the efficacy of low intensity light as an alternative non-invasive cancer treatment.

## Introduction

Conventional methods such as chemotherapy, radiotherapy, and surgery have shown a limited success and effectiveness in treatment of melanoma. According to world health organization the cancer is among the top ten causes of death around the globe and the second cause of death in developed countries [1]. Thus, numerous methods of treatment have been developed and attempted by scientists to combat this modern era disease. Apparently, the primary reason for cancer development is permutations in the DNA of a cell that lead to uncontrolled growth of mutated cells that contribute to tumor growth. Oncogenes are a specific group of growth effectors that promote uncontrolled cell growth and proliferation. These proteins are derived from normal cellular growth effectors (so-called proto-oncogenes) by a limited number of modifications: mutations, insertions, or deletions. Because proto-oncogenes control the cell cycle, it is obvious that should a proto-oncogene be mutated, the potential for an unregulated cell cycle results. This unregulated cell cycle, due to loss of function of tumor suppressor genes and/or increased activity of oncogenes, is the essence of tumor. For therapeutic purposes, there is a growing need to adopt reverse engineering methods, i.e. activation of a certain biological activity that would have detrimental effect on cancer cells.

Application of low intensity light therapy (LILT) towards inducing cellular effects in living organisms has gained widespread popularity to potentially expand the prospect of using this technique for medical applications [2]. To this end, there have been several attempts in recent decade to induce biological function through irradiation of cells/molecules by an external source of light [3–5]. These induced biological functions are the result of energy transferred to the living organism [6–8]. The changes in quantum-mechanical energy states of photosensitive molecules would result in the frequency selectivity of many light induced biological processes [9–11]. Thus, the concept of LILT with an evidence based therapeutic effect in soft tissue and joint repair applications prompted investigation of LILT use in cancer treatment *in vitro* and *in vivo* [4, 12, 13].

Modeling of interactions between external light exposures and living organisms will benefit in optimizing the most effective parameters of applied irradiation. There have been several attempts for computational modeling of influence of irradiation on biological systems [14–16]. However, among them the Resonant Recognition Model (RRM) has demonstrated to be a more accurate technique for computation of frequencies (wavelengths) i.e. f_RRM_, which have resonant effects on proteins biological activity [9, 11, 15]. Since there is an evidence that proteins have certain conducting or semi-conducting properties, a charge moving through the protein backbone and passing different energy stages caused by different amino acid side groups can produce sufficient conditions for a specific electromagnetic radiation or absorption. In our previous research we have shown that such charge transfer through the protein backbone is possible through an excitation process [7, 17].

According to the RRM, a strong linear correlation exists between the predicted and experimentally determined frequencies corresponding to the absorption of electromagnetic radiation of such proteins [18–20]. It is inferred that approximate wavelengths in real frequency space can be calculated from the RRM characteristic frequencies for each biologically related group of sequences. These calculations can be used to predict the wavelength of the light irradiation, λ, that might affect the biological activity of exposed proteins [18–20]. The frequency range predicted for protein interactions is from 10^13^Hz to 10^15^Hz. This estimated range includes IR, visible and UV light. In our previous study the RRM was used to analyze oncogene and proto-oncogene proteins and determine their corresponding characteristic frequencies [19, 20]. These computationally defined RRM frequencies for oncogene (f_1_=0.0302) and proto-oncogene (f_2_=0.0576) proteins can be converted to real space wavelengths of applied irradiation using the ratio λ = 201/f_RRM_. Thus, the computationally predicted wavelength for oncogene activation is 6656nm, and for proto-oncogene is 3490nm.

Of particular interest to this study was irradiation of selected cancer and normal cells with the light of the wavelengths of 3500nm to 6400nm and evaluation of its effects on the cytotoxicity of the selected cells. For this purpose, the LED-based exposure device was designed and developed to operate in the mentioned above wavelengths’ range [21]. There are several theoretical and experimental studies published that used visible and infrared coherent light exposures for various applications [16–20, 22] while, application of far infrared non-coherent light exposures for cancer treatment have not been widely studied.

It was shown that infrared light can penetrate through a human body and tissue can absorb most of its energy in contrary to strong reflection of visible light. Near infrared (NIR) light has maximum depth of penetration in tissue. Within the NIR window, scattering is the most dominant light-tissue interactions which lead to rapid diffusion of propagating light within a tissue. Since scattering increases the distance travelled by photons within tissue, the probability of photon absorption also increases. Because scattering has weak dependence on wavelength, in the NIR wavelength the tissue absorption is limited to light absorption of blood at short wavelengths and water at long wavelengths. As such, the depth of tissue penetration by infrared radiation depends on its wavelengths and can reach a few centimeters with near infrared having the deepest penetration [23, 24]. Hence, the major focus of this study is to investigate the application of infrared exposures for its therapeutic effects on surface or near surface tumors such as breast cancers. In this study, we have used a breast cancer cell line (MCF7) for the *in vitro* evaluation of the effects induced by different light exposures. In particular, we investigated *in vitro* effects of visible light (466- 950nm) and the specific far infrared light wavelengths, 3500– 6400nm (within the range determined computationally by the RRM), on MCF7 and normal dermal epithelial cells used as a control.

## Methods

### Cell Cultures

The Human Breast Cancer cell line (MCF7) and Human Epidermal Melanocytes cell line (HEM) were obtained from the School of Applied Science, RMT University, Australia. Both cell lines were cultured in *Dulbecco's Modified Eagle Medium* (DMEM) (GIBCO, Australia) supplemented with 10% Fetal Bovine Serum (FBS) (Bovogen serum Biologicals, Australia) and 1% penicillin/streptomycin. Cells were incubated at 37°C in a humidified atmosphere containing 5% CO_2_. MCF7 cells were seeded at an initial density of 1 × 10^4^ cells/mL in a *96-well plate* for cell viability analysis, which was conducted by the LDH method following manufacturere's protocol. (Roche Diagnostics, Australia)

### LED-base exposure device

The RRM was employed to computationally determine wavelengths of light that can affect the activity of proto-oncogene and oncogene proteins (3500– 6400nm). Of particular interest was to study the wavelength range that can activate proto-oncogene proteins. Hence, an LED-based exposure system has been developed to emit light at the selected far infrared wavelengths of 3400nm, 3600nm, 3800nm, 3900nm, 4100nm, 4300nm. For the exposure device to operate at the optimal outputs, the input signal of 250mA, 2 kHz and 50% duty cycle has been fed into the system. Adaptor of 12v and 1A current has been fed into circuitry.

In addition, the effects of selected wavelengths of 466nm, 585nm, 626nm (visible light) and 810nm, 850nm and 950nm (near infrared light) have been studied on cancer and non-cancer cell lines. The experimental setup and the exposure system were designed in a way to avoid any cross talk between different LEDs. To make sure that there are no heating effects on irradiated cells, a heat shield material was used between the wells in order to prevent heat dissipation and absorption produced by other LEDs irradiating at the selected wavelengths. In order to achieve a minimum dispersion, the device was designed to have the narrowest possible irradiation angle and the gap between the device and an exposed sample was set at less than 1mm. For the consistency in the experimental setup and the power irradiated at each particular wavelength, all LEDs used in the experiments had the irradiation angle of less than 40° (for minimum power dissipation from the energy source).

Since we aimed at investigating the effects of low intensity light exposures, the optical intensities of LEDs used for exposures were in the range of 15µW to 30µW. The relevance conversion factor between radiant intensity and luminous intensity can be calculated as follows:

K(λ) = I_v_ (Luminous Intensity)/ I_e_ (Radiant Intensity)

### Experimental procedure and setup

Cells were seeded at a density of 1x10^4^ cells /mL in a 96-well plate and incubated overnight. Then, they were treated in triplicate in the same plate. Three variations of exposure and post-exposure regimes were tested on each cell line. Each combination of different exposure and incubation times were repeated three times in order to find the statistically significant results and reveal whether post-exposure incubation or irradiation duration (dose) have any specific effect on cell toxicity.

In first regime the cells were irradiated for 1.5 hours and immediately after the exposure cell viability was tested using LDH or PrestoBlue™ assays without any further post treatment incubation. For the second regime of exposure, cells were irradiated for 1.5 hours and a post treatment incubation for 24 hours at 37°C was performed before LDH or PrestoBlue™ assays. In the third regime, the cells were irradiated for 3 hours and further incubated for 24 hours before LDH or PrestoBlue™ assays. The reason for selecting three different regimes of exposure and incubation was to reveal whether longer incubation or exposure time would induce more significant effects on cell viability/cytotoxicity. These exposure regimes are the optimum exposures that have been optimized through a number of preliminary experimentation with cancer cells.

To eliminate any effects from the heat generated by the IR-LEDs used in the exposure device, we introduced a heat shield gel (Inventables, USA). The gel was placed around the wells in the 96-well plate from outside and between the gaps. Plates were placed in a UV camera two times for 30 min before seeding. More importantly, to eliminate any cross talk between the LEDs and the effect of two frequencies on the same well, we had left wells empty around each well where the experiments were run.

### Phase Contrast Microscopy

The effects of irradiation on human breast cancer and normal cells morphology have been studies by phase contrast microscopy. Cells were seeded at a density of 2 × 10^5^ cell/mL in a 24-well plate and incubated overnight. The next day, cells were exposed to near-infrared, far-infrared and visible light irradiation for 3 hours, and then further incubated for 24 hours. After 24 hours of incubation, they were washed carefully with phosphate-buffered saline (PBS). Phase contrast microscopy images for each wavelength were taken at 100X magnification using Nikon Eclipse Ti-E microscope (Nikon Instruments Inc, Japan).

### PrestoBlue™ Cell Viability assay

PrestoBlue™ assay was used as reagent for rapidly evaluating the viability and proliferation of MCF7 for three different regimes of exposure and post exposure incubation. The exposures were conducted for 6 selected LEDs in far infrared wavelength range that is computationally calculated by RRM model. PrestoBlue™ quantitatively measured any variation in cell viability of breast cancer cells as a result of these wavelengths exposure. Cells were seeded at a density of 1x10^4^ cells /mL in a 96-well plate and incubated overnight before the exposure. The experiments were conducted in triplicated and repeated three times. The procedure and preparation to evaluate cell viability by PrestoBlue™ were closely followed by the accompanied manual (Invitrogen Technologies, Australia). Then, the result were measured at 595nm using a 96 well ELISA plate reader (Thermo Electron Corporation, USA). The control was the mean absorbance from untreated cells and the background was just the cell culture media without cells.

### Lactate Dehydrogenase (LDH) assay

LDH assay was used to measure and compare the effects of different exposure regimes of visible, near infrared and far infrared wavelengths on human breast cancer and normal cells. This standard quantitative assay measures the amount of Lactate dehydrogenase activity released in the media as a result of our experiment. The effects of light exposures on breast cancer and normal cells were quantitatively determined. Cells were seeded at a density of 1x10^4^ cells /mL in a 96-well plate and incubated overnight before the exposure. The cells were exposed in triplicate for each wavelength of applied irradiation. Three different regimes of exposure and post-exposure incubation were selected for evaluation. The experiments were repeated three times with MCF7 and HEM cells. The LDH released from damaged cells resulted from the exposures was measured by the Cytotoxicity Detection Kit (Roche Diagnostics, Australia) according to the manufacturer's instructions. The absorbance of the color generated was measured at 495 nm using a 96 well ELISA plate reader (Thermo Electron Corporation, USA). The background control (medium only) values were subtracted from each studied well. Low control was the mean absorbance from untreated cells (basal release of LDH) and high control or positive control was the mean absorbance from lysis cells (maximum release of LDH). Cytotoxicity percentage for each cell line was calculated according to the following equation:

Cytotoxicity = 100 × [(experimental value – low control)/(high control – low control)]

In addition, one-way ANOVA test was used as the statistical analysis technique for the numerical results obtained from OD reading as a result of LDH assay implementation.

## Results and Discussion

### Morphological examination of cellular toxicity

The effects of far infrared exposures on morphological features in MCF7human breast cancer cells were examined using phase contrast microscopy. The images were taken from MCF7 cells with a density of 2x10^5^ cells/ mL and seeded overnight in a 24-well plate. The day after the seeding of the cells, the plate was irradiated for 3 hours and incubated for the next 24 hours. Just before imaging, these wells were washed carefully with PBS in order to remove any detached or floating cells in the medium.

**Figure 1:**
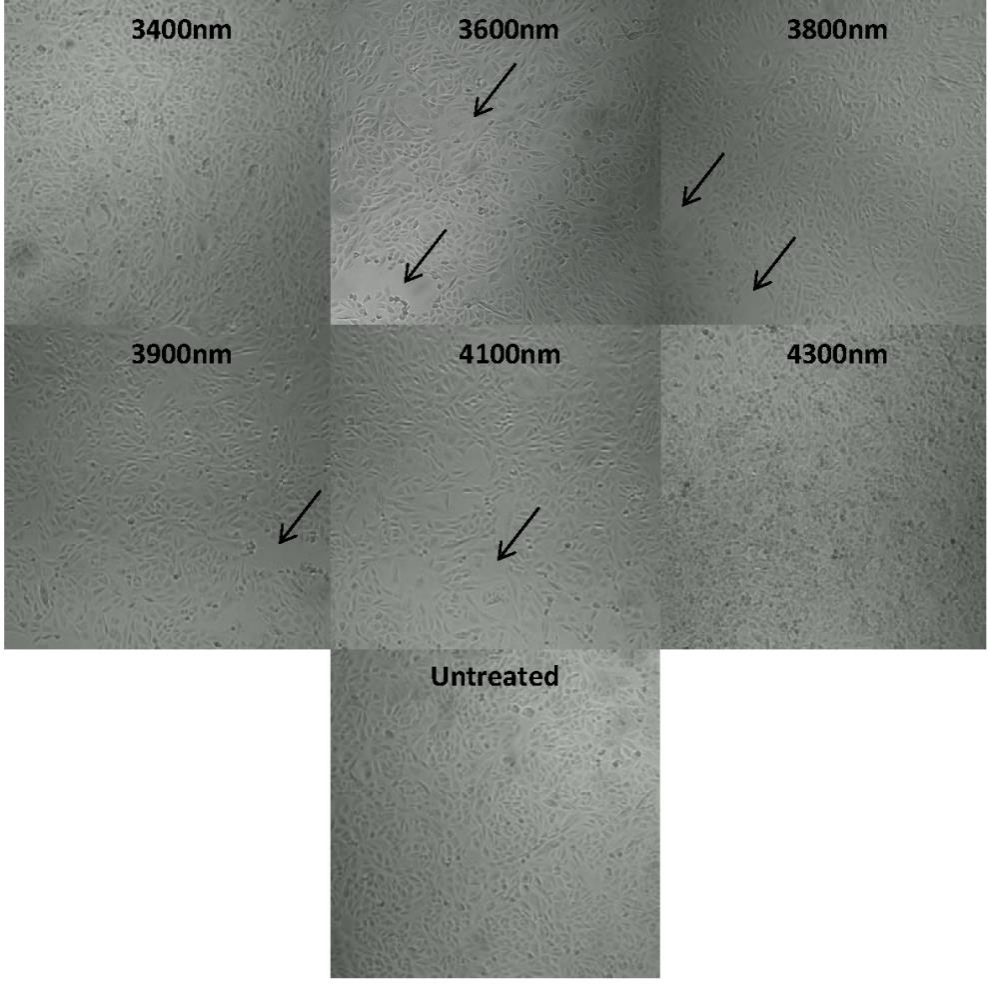
Phase contrast microscopy images of far infrared (3400nm, 3600nm, 3800nm, 3900nm, 4100nm, 4300nm) exposures on MCF7 cells along with untreated MCF7. Arrow heads indicate the areas of cell death due to exposure. The images demonstrate MCF7 cells after 3 hours of far infrared exposure and 24 hours of post exposure incubation.

As it is apparent from Fig. 1, there are clearly visible cell detachment areas (indicated by arrows). When comparing the images obtained from the exposed well to the unexposed specimen, it can be inferred that irradiation with wavelength of 3400nm, 3600nm, 3800nm, 3900nm, 4100nm and 4300nm induced detrimental effects on MCF7 cells. Among the entire far infrared light range used for exposures, the wavelengths 3600nm, 4100nm, and 4300nm induced more significant cell detachment from the adherent confluent layer than other studied wavelengths. Of note, the untreated cells (unexposed) do not show any changes in their morphology or cytotoxicity.

### Quantification of cell viability from PrestoBlue™ Assay

PrestoBlue™ cell viability assay was used to analyze the effects of different regimes of exposures and post exposure incubation on MCF7 cancer cell line. The evaluation of cell viability was conducted on MCF7 cells exposed at the far infrared wavelengths predicted computationally.

**Figure 2:**
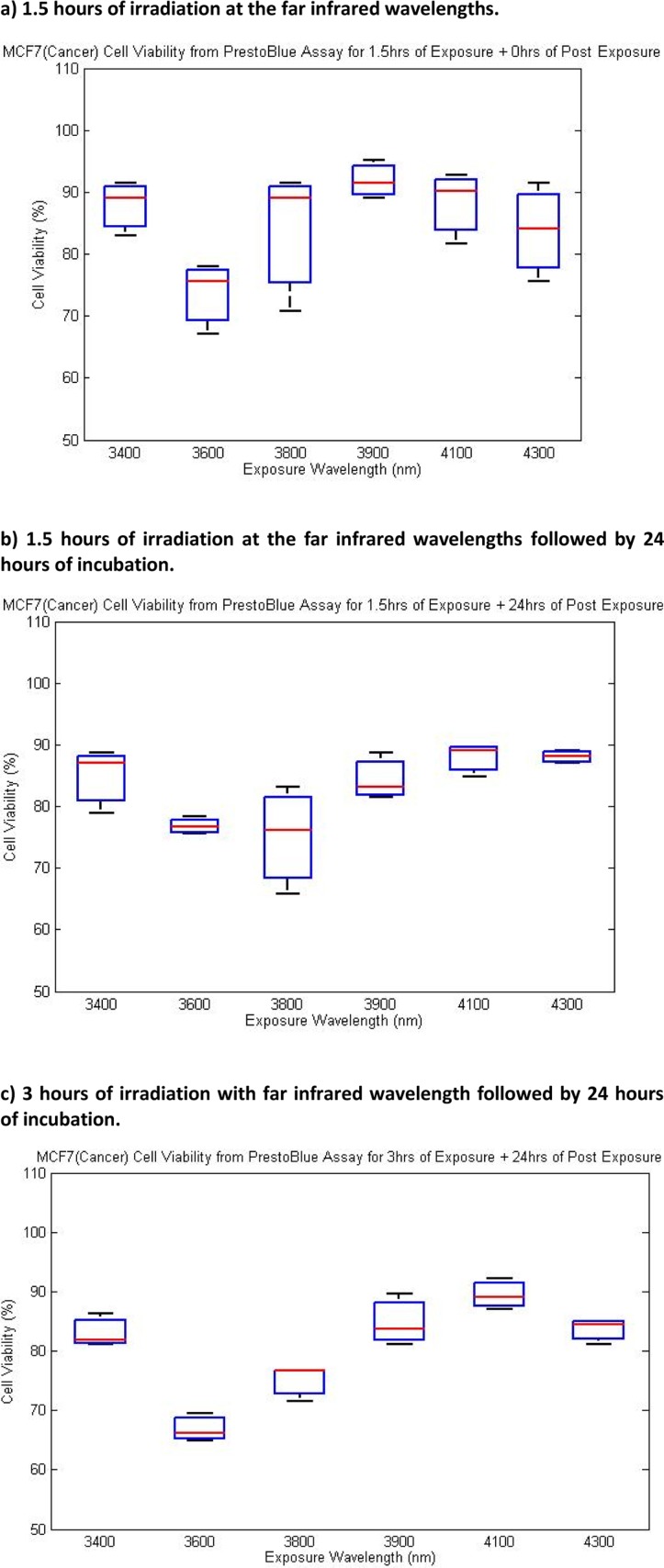
Effect of different exposure regimes and post exposure incubation on cell viability of MCF7 measured by PrestoBlue™ cell viability assay. OD was measured at 595 nm after addition of the PrestoBlue™ reagent and incubation for 30 min.

As can be seen from Fig. 2a) there is at least 10% decrease in cell viability of MCF7 exposed by 1.5 hours of far infrared exposures. Exposure at wavelength of 3600nm led to more than 20% reduction in cell viability. Fig. 2b) shows PrestoBlue™ cell viability measurement after 1.5hours of irradiation at far infrared wavelengths followed by 24 hours of incubation. The pattern/trend is consistant with the results shown in Fig. 2a) where we have had greater than 10% decrease in cell viability as a result of exposure. The 24 hours of incubation did not induce significant changes in the cell viability compared to Fig. 2a). Fig. 2c) presents PrestoBlue™ assay results after 3 hours of exposures followed by 24 hours of incubation. This result is in agreement with the result obtained within radiation regimes shown in Fig. 2a and 2b. The induced effects the regime 2c are more significant than that of exposure regimes presented in 2a and 2b.

As it is evident from the results shown in Fig. 2, cell viability measurements of different exposure regimes reveal that 3 hours of exposure followed by 24 hours of incubation has the highest overall impact on MCF7 cancer cell line. Comparison of results presented in Fig. 2a), 2b), and 2c) clearly indicates that infrared light exposures have increased cell apoptosis (cell death).

### Quantification of LDH activity from damaged cells

LDH assay was used to evaluate the irradiation effect of far infrared, near infrared, and visible light exposures on MCF7 human breast cancer cells and HEM human normal cells. These analyses have been conducted in different regimes of exposure and incubation and for a range of wavelengths in the far infrared, near infrared and visible light in order to comprehend and distinguish between the induced effect of these exposures on MCF7 and HEM cells. Three different regimes of exposures and post-exposure incubations have been proposed to reveal any significant effect that irradiation has over post-exposure incubation or *vice versa*.

**Figure 3:**
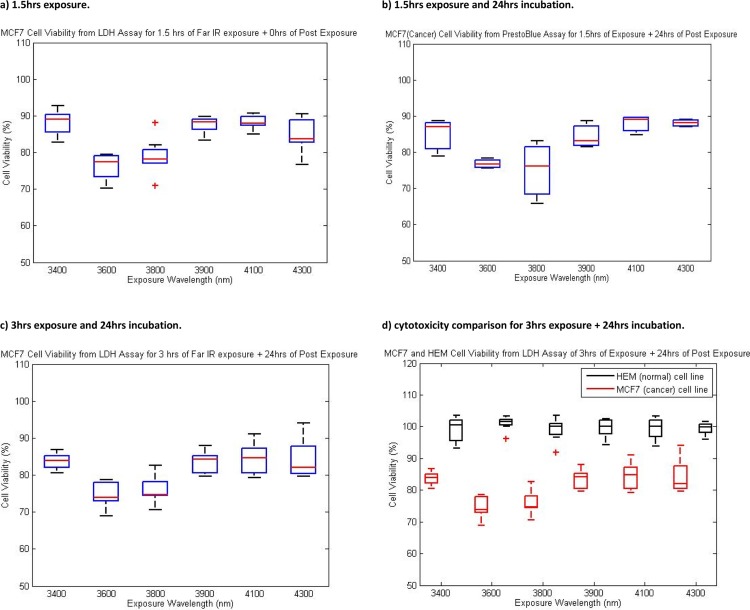
Cell viability in response to different regimes of exposure and post-exposure. Part a), b) and c) demonstrate cytotoxic effect of exposure regime of one, two and three respectively on MCF7. Part d) shows cytotoxic effect of third regime of exposure on MCF7 (red box) and HEM (black box).

The graphs presented in Fig. 3 indicate clearly the changes in cell viability of MCF7 induced by different exposures to far infrared light. OD was measured at 492 nm after the completion of LDH procedures. From Fig. 3a) it can be inferred that upon exposure at the studied far infrared wavelengths cell viability was reduced by about 10% across all wavelengths. Interestingly, the second experimental regime, 1.5 hours of exposure along with 24 hours of incubation (shown in Fig. 3b), was comparable with the viability measured by PrestoBlue™ after 1.5 hours of exposure and the 24 hours of post exposure incubation (Fig. 2a) and did not result in the significant increase in cell death. The cell viability measurement of first regime of exposure which does not have post exposure incubation compared with the second regime that has 24 hours of post exposure incubation confirms the fact that the cytotoxic effect of exposure is not temporary as decrease in cell viability is much more prominent than cell recovery. From Fig. 3c it is evident that the exposure at the wavelength of 3600nm induced more cytotoxic effect in MCF7 cells than other far-infrared exposures. Within the RRM analysis, it was computationally predicted that the wavelength of 3500nm would induce effect on oncogene proteins which in turn as expected will reduce the viability of MCF7 human breast cancer cells. Fig. 3d) provides data for comparison of the cytotoxic effects of third exposure regime (3hrs exposure and 24hrs of incubation) at selected far infrared wavelengths induced in MCF7 human breast cancer cell line (in red color) and HEM normal human cell line (in black color). As it can be seen, the cytotoxic effect of far infrared exposure on MCF7 is significant when compared to invisible effect of the same exposure regime on normal human cell line.

**Figure 4:**
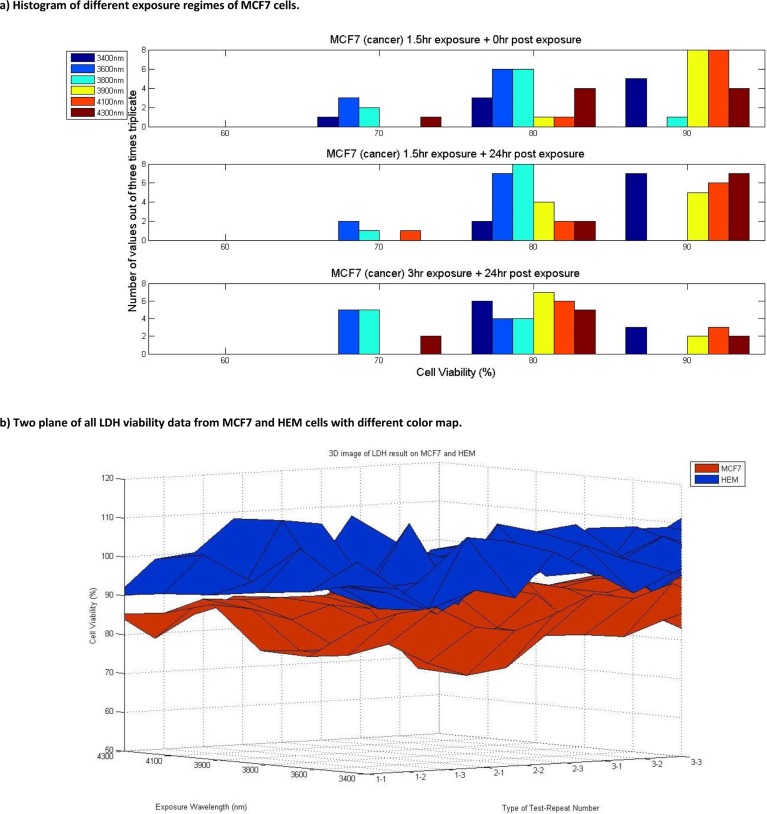
Comparison of different regime of irradiation on MCF7 and HEM cells. Part a) provides histograms of all MCF7 cell viability changes for different regime of exposure. Comparison of all figure demonstrate the significant cell viability shift from 90% on the top figure to 70-80% on the bottom figure. Part b) shows two plane of blue and red for HEM and MCF7 respectively. Each surface shows three times triplicate of LDH analysis for 3 different regime of far infrared exposure.

As it can be seen from Fig. 4a), 3 hours exposure along with 24 hours of incubation induced the most cytotoxic effect on MCF7 cells compared to the other two exposure regimes. It can be observed that for 1.5 hours of exposure cell viability of MCF7 is around 90%, while by exposing MCF7 cells using the second regime of exposure the cell viability was reduced down to 80%. The third regime of exposure (3 hours of exposure along with 24 hours of incubation) resulted in further reduction of cell viability down to 70 percent. Fig. 4b) clearly shows the plane of data from all regimes of exposure at all far infrared wavelengths of irradiation of MCF7 and HEM cells. The graph reveals a clear cytotoxic effect observed for cancer cells while normal cells viability is balanced around 100% which means there is no effect caused by irradiation. From Fig. 4a) and 4b) can be seen that these two planes are overlapping at some point in the first experimental regime but in general, these two planes clearly show that light exposures produced cytotoxic effects on cell viability of MCF7 cells. The results reveal that the cell viability of MCF7 (red) is hovering between 70 and 90 percent, while the cell viability of HEM cells (blue) is hovering between 90 and 100 percent. The graph in Fig. 4b) indicates clear effect of far infrared exposure on MCF7 compared to HEM.

**Figure 5:**
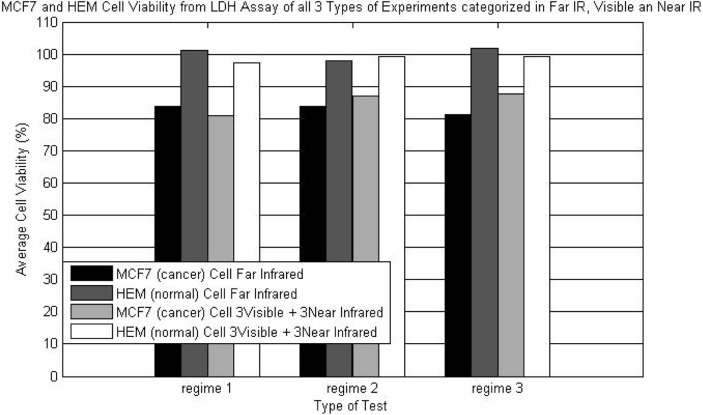
Cytotoxic effect of different exposure regime of MCF7 cells compared to HEM cells at the selected wavelengths in far infrared, near infrared and visible light range.

Fig. 5 shows the effects of different exposure regimes and different wavelength range on MCF7 and HEM cells. From this figure we can observe that the selected wavelengths in far infrared range induced the most cytotoxic effect on cancer cells. In addition, it is shown that MCF7 cells are affected by the exposures at near infrared wavelengths which require further investigation and analysis. In fact, the results shown in Fig. 5 reconfirm the expected cytotoxic effect of computed far infrared wavelengths on MCF7 cells.

## Conclusions

The RRM was used to predict computationally the frequencies (wavelengths) of light radiation that can affect the biological functions of oncogenes and proto-oncogene proteins. Low intensity light exposures in the wavelength range of 3500nm to 6400nm was predicted to have effect on cancer cell growth [20]. Our previous experiments on murine melanoma (B16F10) and Chinese Hamster Ovarian (CHO) cell lines, demonstrated a significant increase in cytotoxic activity as a result of far infrared light exposures [25]. In this study, we evaluated the detrimental effect of the RRM computed far infrared wavelengths on MCF7 human breast cancer cells and HEM normal human cells. For comprehensive assessment of the effects of light exposures, the comparison between the induced effects in MCF7 and HEM cells by exposure of near infrared and visible light range were conducted and demonstrated.

The results obtained from phase contrast microscopy and two quantitative assays of LDH and PrestoBlue™ reveal that far infrared exposures have more significant impact on MCF7 cells while the viability of normal cells has not been affected by the exposures. In addition, among the entire range of wavelengths proposed by the RRM, the wavelength of 3500nm was determined as the activation wavelengths of proto-oncogene proteins [20]. It was expected that the light exposure at this particular wavelength will cause a decrease in cell viability of cancer cells. Throughout our experimental evaluation we showed that the maximum cytotoxicity is achieved at the exposure of 3600nm. This result requires further investigation via more in-depth study such as advanced molecular biology and microscopy techniques to corroborate this finding. These further analyses of the result are in implementation and preparation phase for our next publication.
